# Role of pathophysiology of patellofemoral instability in the treatment of spontaneous medial patellofemoral subluxation: a case report

**DOI:** 10.1186/1752-1947-4-148

**Published:** 2010-05-22

**Authors:** Ertuğrul Akşahin, Feridun Yumrukçal, Halil Yalçın Yüksel, Dağhan Doğruyol, Levent çelebi

**Affiliations:** 1 Ankara Numune Education and Research Hospital, Ankara, Turkey; 2 Department of Orthopedics and Traumatology, Erzincan State Hospital, Erzurum, Turkey

## Abstract

**Introduction:**

Medial patellar subluxation is usually seen after lateral retinacular release. Spontaneous medial subluxation of the patella is a very rare condition. There are few reports in the literature on the pathophysiology of iatrogenic medial patellar subluxation. To our knowledge, there are no reports of the pathophysiology of non-iatrogenic medial patellar subluxation in the English literature. In this study we present a case of spontaneous medial patellar instability that is more prominent in extension during weight bearing. We also try to define the treatment protocol based on pathophsiology.

**Case presentation:**

We report the case of a 21-year-old Turkish man with spontaneous medial patellar instability. He had suffered right knee pain, clicking and popping sensation in the affected knee for three months prior to presentation. Clinical examination demonstrated medial patellar subluxation that is more prominent in extension during the weight bearing phase of gait and while standing. Increased medial tilt was observed when the patella was stressed medially. Conventional anterior to posterior, lateral and Merchant radiographs did not reveal any abnormalities. After three months of physical therapy, our patient was still suffering from right knee pain which disturbed his gait pattern. Throughout the surgery, medial patellar translation was tested following the imbrication of lateral structures. He still had a medial patellar translation that was more than 50% of his patellar width. Patellotibial ligament augmentation using an iliotibial band flap was added. When examined after surgery, the alignment of the patella was effectively corrected.

**Conclusions:**

Chronic imbalance between the strengths of vastus lateralis and vastus medialis results in secondary changes in passive ligamentous structures and causes additional instability. Physical therapy modalities that aim to strengthen the vastus lateralis might be sufficient for the treatment of spontaneous medial instability. There would be no need for any surgical intervention if spontaneous medial instability was recognized before the additional instability occured. If necessary, lateral imbrication followed by lateral patellotibial ligament augmentation can be performed, and these would effectively correct spontaneous medial patellofemoral instability.

## Introduction

Medial patellar subluxation is a disabling clinical entity that is usually seen after surgical procedures performed for lateral patellofemoral instability. These procedures are lateral retinacular release [1-6] and medial transfer of tibial tubercle [7]. Medial patellar subluxation without any previous surgical procedure is extremely rare. We detected 163 reported cases of medial patellar subluxation in the English literature. Eight cases were of post-traumatic patients and only five were spontaneous, while the remaining cases were secondary to previous surgical procedure [1,4,8].

Although few surgical methods such as direct retinacular repair [1,4], reconstruction of patellotibial ligament using iliotibial band, patellar tendon or fascia lata [4,9] and arthroscopic medial retinacular release [8] are suggested methods for the treatment of medial patellar instability, etiology was not taken into consideration in any of these studies. The choice of surgical method for different pathophysiologies, therefore, has yet to be defined.

Here we present a case of spontaneous medial patellar subluxation that is more prominent in extension while weight bearing.

## Case presentation

**Figure 1:**
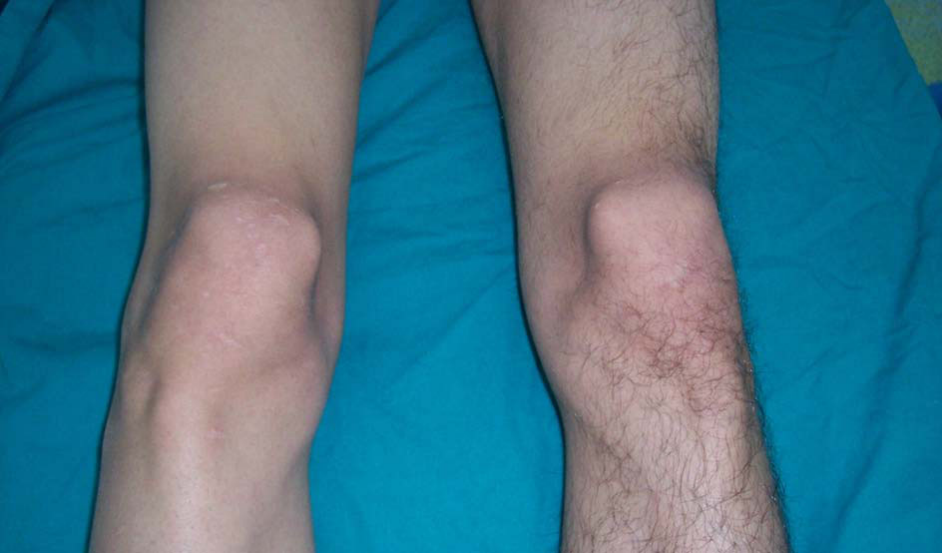
**The patellofemoral relationship of our patient at supine position**.

**Figure 2:**
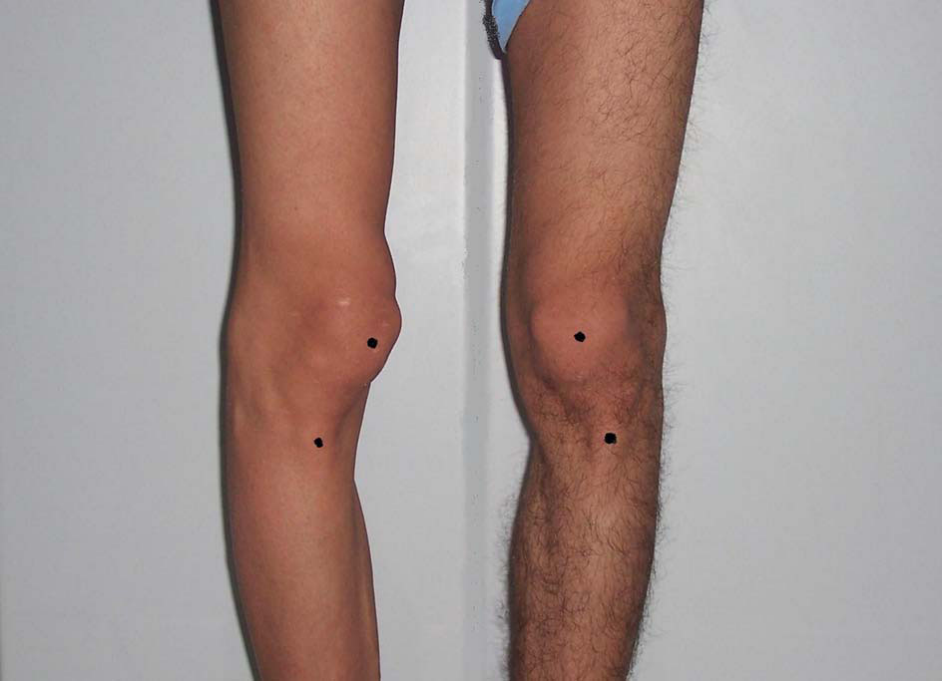
**Medial subluxation of the right patella was more prominent while bearing weight**.

A 21-year-old Turkish man was admitted to our clinic for right knee pain, clicking and popping sensation in the affected knee for three months prior to his presentation. Difficulties in climbing stairs and disability during sports activities were the other symptoms of our patient. He reported no previous physical or surgical treatment for any other knee pathology and did not have any known history of trauma. On our patient's physical examination, atrophy of the quadriceps muscle was observed. Medial patellar apprehension test was positive and increased passive medial patellar mobility was observed when his patella was stressed medially. His gravity subluxation test was negative. His physical examination also revealed that medial subluxation of his right patella was more prominent in extension while weight bearing (Figures 1 and 2). We also noted patellofemoral hypermobility. He had no complaints related with his left knee. Conventional anterior to posterior, lateral and Merchant radiographs of our patient did not reveal any abnormality.

Our patient was initially treated with physical therapy. A quadriceps strengthening rehabilitation program and neuromuscular stimulation focused on the vastus lateralis muscle and was continued for three months. After three months of rehabilitation, some improvement was achieved in his quadriceps strength and the medial translation of his patella was less than the pre-treatment level. However, he still suffered from right knee pain, which disturbed his gait pattern. As such, surgery was planned.

Using a lateral parapatellar approach, we first performed direct lateral retinacular imbrication. We evaluated provocative patellofemoral tracking during flexion and extension of the knee by pressing on the inferior lateral pole of his patella. The achieved patellar stability was not sufficient and his patella was still moving medially more than 50% of its width. We dissected a strip of his iliotibial band approximately 1 cm wide and 4 cm in length, thus leaving distal base of the strip attached to Gerdy's tubercle. Using this strip, we augmented the patellatibial ligament. As the medial subluxation of his patella was more prominent in extension, we tensioned the strip in extension.

Post-operatively our patient's knee was immobilized using a brace for six weeks. He was allowed to walk with partial weight bearing after the first post-operative day until the fourth post-operative week. During the first week he was allowed to perform passive knee extension and active assistive flexion exercises within a range of 0 to 90°. Full active range of motion exercises and full weight bearing was allowed after the third post-operative week. Quadriceps strengthening physical therapy focused on the vastus lateralis with neuromuscular stimulator was continued for three months after the operation. A home exercise program was also given to our patient.

He was examined weekly for the first month. Follow-up visits were made by phone interview monthly and by clinical examination at intervals of three months. By the end of the sixth week of the surgery he achieved full range of motion. Meanwhile, full quadriceps strength was achieved at the end of the third post-operative month. Our patient did not experience any patellofemoral instability during his follow up examinations.

During his last visit for follow-up examination, atrophy of his quadriceps muscle was completely resolved and both medial patellar apprehension and passive medial patellar mobility tests of our patient were negative. Gravity subluxation test was also negative. He was evaluated subsequently according to the clinical score and pain scale defined by Hughston *et al*. [4] (Table 1). Our patient's pre-operative functional level limited performance of his daily activities. At the end of the first year, however, he was categorized as vigorous recreational. While he used to have severe pain, he described mild pain with competitive sports in the post-operative period and no pain with daily activities.

**Table 1:** Functional level and intensity of pain reported by patients undergoing surgical correction of medial subluxation of the patella (data from Hughston [5]).

Functional level	Pain
External support required or sedentary activities	None
Activities of daily living	Mild
Light recreational	Moderate
Moderate recreational	Severe
Vigorous recreational	Totally disabling
Competitive	
Highly competitive	

## Discussion

Debates on the diagnosis and treatment of medial patellar subluxation have been ongoing since it was first described by Hughston and Deese [3] in patients who have undergone lateral retinacular release. Iatrogenic medial patellar subluxation following lateral retinacular release is a rare entity and non-iatrogenic medial patellar dislocation, whether traumatic or spontaneous, is even more uncommon. Among the 163 cases of medial patellar subluxation reported in the literature [8], only five were spontaneous [1,4,8].

However, in the study conducted by Shellock *et al*. [10], patellofemoral tracking had been evaluated by kinematic magnetic resonance imaging (MRI) in 130 patients with symptomatic patellofemoral joint. The authors discovered that there was medial patellar subluxation in 106 of the patients (41%). Thus they concluded that the non-iatrogenic medial patellofemoral subluxation is a far more common clinical entity, but has not been appreciated in patients with knee pain.

On the other hand, the studies on the pathophysiology of iatrogenic medial subluxation of the patella revealed that detachment of vastus lateralis from the patella is the most encountered reason [3,11]. Nonweiler and De Lee [11] analyzed five patients with positive gravity subluxation test, which indicates that vastus lateralis is not functioning properly. They concluded that the main reason for medial patellar subluxation following lateral release was vastus lateralis insufficiency. In contrast to this common opinion about the etiopathology of medial instability of patients who underwent lateral release, Quervain *et al*. [12] claimed that medial patellofemoral instability is mostly caused by the insufficiency of the lateral retinaculum following the release of the patellotibial and the epicondylopatellar ligaments. The authors evaluated two patients suffering from medial subluxation who had undergone lateral release with gait analysis, and they suggested that subluxation was becoming more prominent in the unloading phase of gait when the quadriceps was inactive.

We found nothing in the literature that describes the pathophysiology of spontaneous medial patellar instability. Although there is no satisfactory information, the treatment algorithm is almost the same with iatrogenic and traumatic group. In our case we observed that medial subluxation was more prominent during the weight bearing phase of gait when our patient's quadriceps were active. Additionally, during surgery, we did not observe any problem with vastus lateralis integrity. In our opinion, rather than the imbalance between medial and lateral passive structures of the knee joint, long-term overdominancy of vastus medialis to vastus lateralis was probably playing a major role in the pathophysiology of medial subluxation as in our the case of our patient.

Because the pathophysiology of iatrogenic and spontaneous medial patellar subluxation varies, rehabilitation program should also vary. Physical therapy focused on the quadriceps muscle strengthens both the vastus lateralis and medialis muscles. Although the imbalance between these two muscles may be lessened it would not be eliminated. For this reason we additionally applied electrical stimulus to vastus lateralis muscle for three months pre-operatively. At the end of the third month, although our patient had already achieved some improvement in his quadriceps strength and medial translation of patella he was still suffering from right knee pain which disturbed his gait pattern. We thought that the reason for medial subluxation was not only the vastus medialis overdominancy, but also the insufficiency of lateral passive structures due to irreversible elongation of the ligaments as a result of this chronic imbalance of the active structures. Furthermore, we speculate that the misdiagnosed patients with occult medial patellar instability described by Shellock *et al*. [10] are probably the precursors of advanced stage medial patella subluxation and dislocations. As available physical therapy measures were not sufficient to restore the balance between the ligamentous structures, we planned surgery to reconstruct these structures.

Different methods have been published for the surgical treatment of medial instability [1,4,7-9,11]. Hughston *et al*. [4] published a report on surgically treated 65 knees with medial instability. They have performed direct repair of the lateral retinaculum in 39 patients, and the remaining 26 were treated with the reconstruction of patellotibial ligament, of which 12 were by the help of iliotibial band and 14 were by the help of patellar tendon. They concluded that the reconstruction of lateral patellotibial ligament effectively corrected medial instability. Recently, Shannon and Keenel [8] reported satisfactory results of arthroscopic medial retinacular release for medial instability of patella. Two of the cases they described were spontaneous. They emphasized that arthroscopic medial patellar release was a suitable method of treating spontaneous medial patellar instability but also noted that this method should not be used in hypermobile patella. In another study conducted by Richman and Scheller [1], a patient with spontaneous medial instability was successfully treated with imbrication of the lateral retinaculum.

In our case medial translation of patella was more than 50% when medial patellar laxity test was performed, thus indicating a hypermobile patella. We thus preferred to utilize an open surgical technique. Following the imbrication of lateral structures, medial patellar translation was tested and it was still more than 50%. As a result, we decided to augment the insufficient lateral structures of our patient. We performed patellotibial ligament augmentation using an iliotibial band flap described by Hughston *et al*. [4] and successfully restored our patient's patellofemoral stability. At the end of the treatment period he could already return to his daily activities.

## Conclusions

Spontaneous medial patellar instability is a subtle clinical entity caused by an imbalance between the strength of vastus lateralis and vastus medialis. Physical therapy modalities aiming to strengthen the vastus lateralis might be enough for the treatment of spontaneous medial instability without necessitating any surgical intervention. This would only be possible, however, if spontaneous medial instability is diagnosed before additional instability occurs in the lateral patellar retinacular structures due to chronic overlengthening. If surgery is indicated due to these secondary changes in the passive structures, lateral imbrication followed by an additional lateral patellotibial ligament augmentation if necessary, can be performed. These would effectively correct spontaneous medial patellofemoral subluxation. Data from larger case series are needed to define the pathophysiology of medial patellofemoral instability and the different etiologies and also to treat patients appropriately.

## Competing interests

The authors declare that they have no competing interests.

## Authors' contributions

EA and FY analyzed and interpreted our patient's data, performed the surgery and contributed in writing the manuscript. HYY and LC drafted the manuscript and revised it critically for important intellectual content. DD participated in the medical interventions, took the photographs, and undertook the literature review. All authors read and approved the final manuscript.

## Consent

Written informed consent was obtained from our patient for publication of this case report and any accompanying images. A copy of the written consent is available for review by the Editor-in-Chief of this journal.
